# SIRT1 Promotes N-Myc Oncogenesis through a Positive Feedback Loop Involving the Effects of MKP3 and ERK on N-Myc Protein Stability

**DOI:** 10.1371/journal.pgen.1002135

**Published:** 2011-06-16

**Authors:** Glenn M. Marshall, Pei Y. Liu, Samuele Gherardi, Christopher J. Scarlett, Antonio Bedalov, Ning Xu, Nuncio Iraci, Emanuele Valli, Dora Ling, Wayne Thomas, Margo van Bekkum, Eric Sekyere, Kacper Jankowski, Toby Trahair, Karen L. MacKenzie, Michelle Haber, Murray D. Norris, Andrew V. Biankin, Giovanni Perini, Tao Liu

**Affiliations:** 1 Children's Cancer Institute Australia for Medical Research, Randwick, Australia; 2 The Centre for Children's Cancer and Blood Disorders, Sydney Children's Hospital, Randwick, Australia; 3 Department of Biology, University of Bologna, Bologna, Italy; 4 Garvan Institute of Medical Research, Darlinghurst, Australia; 5 Fred Hutchinson Cancer Research Centre, University of Washington, Seattle, Washington, United States of America; Beckman Research Institute, City of Hope, United States of America

## Abstract

The N-Myc oncoprotein is a critical factor in neuroblastoma tumorigenesis which requires additional mechanisms converting a low-level to a high-level N-Myc expression. N-Myc protein is stabilized when phosphorylated at Serine 62 by phosphorylated ERK protein. Here we describe a novel positive feedback loop whereby N-Myc directly induced the transcription of the class III histone deacetylase SIRT1, which in turn increased N-Myc protein stability. SIRT1 binds to Myc Box I domain of N-Myc protein to form a novel transcriptional repressor complex at gene promoter of mitogen-activated protein kinase phosphatase 3 (MKP3), leading to transcriptional repression of MKP3, ERK protein phosphorylation, N-Myc protein phosphorylation at Serine 62, and N-Myc protein stabilization. Importantly, SIRT1 was up-regulated, MKP3 down-regulated, in pre-cancerous cells, and preventative treatment with the SIRT1 inhibitor Cambinol reduced tumorigenesis in *TH*-*MYCN* transgenic mice. Our data demonstrate the important roles of SIRT1 in N-Myc oncogenesis and SIRT1 inhibitors in the prevention and therapy of N-Myc–induced neuroblastoma.

## Author Summary

The class III histone deacetylase SIRT1 is repressed by tumor suppressor genes and exerts divergent effects on tumorigenesis depending on its down-stream targets. Small molecule SIRT1 inhibitors have shown promising anti-cancer effects both *in vitro* and *in vivo*. Here we identified SIRT1 as a gene directly up-regulated by N-Myc and identified SIRT1-mediated transcriptional repression as a novel mechanism responsible for maintaining N-Myc oncoprotein stability. Moreover, SIRT1 contributed to N-Myc–induced cell proliferation, and preventative treatment with the SIRT1 inhibitor Cambinol reduced tumorigenesis in N-Myc transgenic mice. Our data identify SIRT1 as an important co-factor for N-Myc oncogenesis and provide important evidence for the potential application of SIRT1 inhibitors in the prevention and therapy of N-Myc–induced neuroblastoma.

## Introduction

Neuroblastoma, which originates from precursor neuroblast cells, is the most common solid tumor in early childhood. MYCN oncogene amplification and consequent N-Myc mRNA and protein over-expression, are seen as a clonal feature in a quarter of tumors, and correlate with poorer prognosis in patients with neuroblastoma [1], [2].

Myc oncoproteins, including N-Myc and c-Myc, induce malignant transformation by binding to cognate DNA sequences and modulating gene transcription, leading to cell proliferation [3]. Stabilization and degradation of Myc oncoproteins are controlled by ordered phosphorylation at two specific sites: Serine 62 (S62) and Threonine 58 (T58). While T58 phosphorylation promotes Myc protein ubiquitylation and degradation through the 26S proteasome-mediated proteolysis, S62 phosphorylation stabilizes Myc proteins [4]–[6]. One of the key factors which promote Myc protein phosphorylation at S62 is extracellular signal-regulated protein kinase (ERK) [4].

Recruitment of histone deacetylase (HDAC) proteins to gene promoters induces histone hypo-acetylation and transcriptional repression, particularly of tumor suppressor genes [7]. Gene expression and deacetylase activity of the class III HDAC SIRT1 are often altered in human cancer tissues (reviewed in [8]). SIRT1 is up-regulated in poorly differentiated adenocarcinomas, compared with normal counterparts, in three transgenic mouse models of prostate cancer and in human prostate tumor tissues [9]. SIRT1 is also over-expressed in human gastric cancer tissues, and SIRT1 over-expression correlates with advanced disease stage, tumor metastasis and poor patient prognosis [10]. Paradoxically, SIRT1 expression is reduced in human colon cancer tissues in general [11], but significantly over-expressed in human colon cancer tissues associated with microsatellite instability and CpG island methylator phenotype [12].

SIRT1 induces histone deacetylation and methylation [13], [14], promoter CpG island methylation [15], transcriptional repression of tumor suppressor genes [16], and deacetylation of tumor suppressor proteins [17], [18]. SIRT1 may therefore play a critical role in tumor initiation and progression by blocking apoptosis and/or promoting cell growth. On the other hand, by deacetylating catenin and survivin, SIRT1 can block cell proliferation and promote apoptosis [19], [20].

In the current study, we have identified two Myc-responsive element E-Boxes at the SIRT1 gene core promoter, and shown that N-Myc up-regulated SIRT1 gene transcription. In a positive feedback loop, SIRT1 binds to Myc Box I domain of N-Myc protein to form a novel transcriptional repressor complex at the gene promoter of mitogen-activated protein kinase phosphatase 3 (MKP3), leading to transcriptional repression of MKP3, ERK protein phosphorylation, N-Myc protein phosphorylation at Serine 62 and N-Myc protein stabilization. These mechanisms contributed directly to the initiation and progression of N-Myc-driven oncogenesis in a murine model of neuroblastoma.

## Results

### Transcriptional up-regulation of SIRT1 by N-Myc promotes neuroblastoma cell proliferation

**Figure 1:**
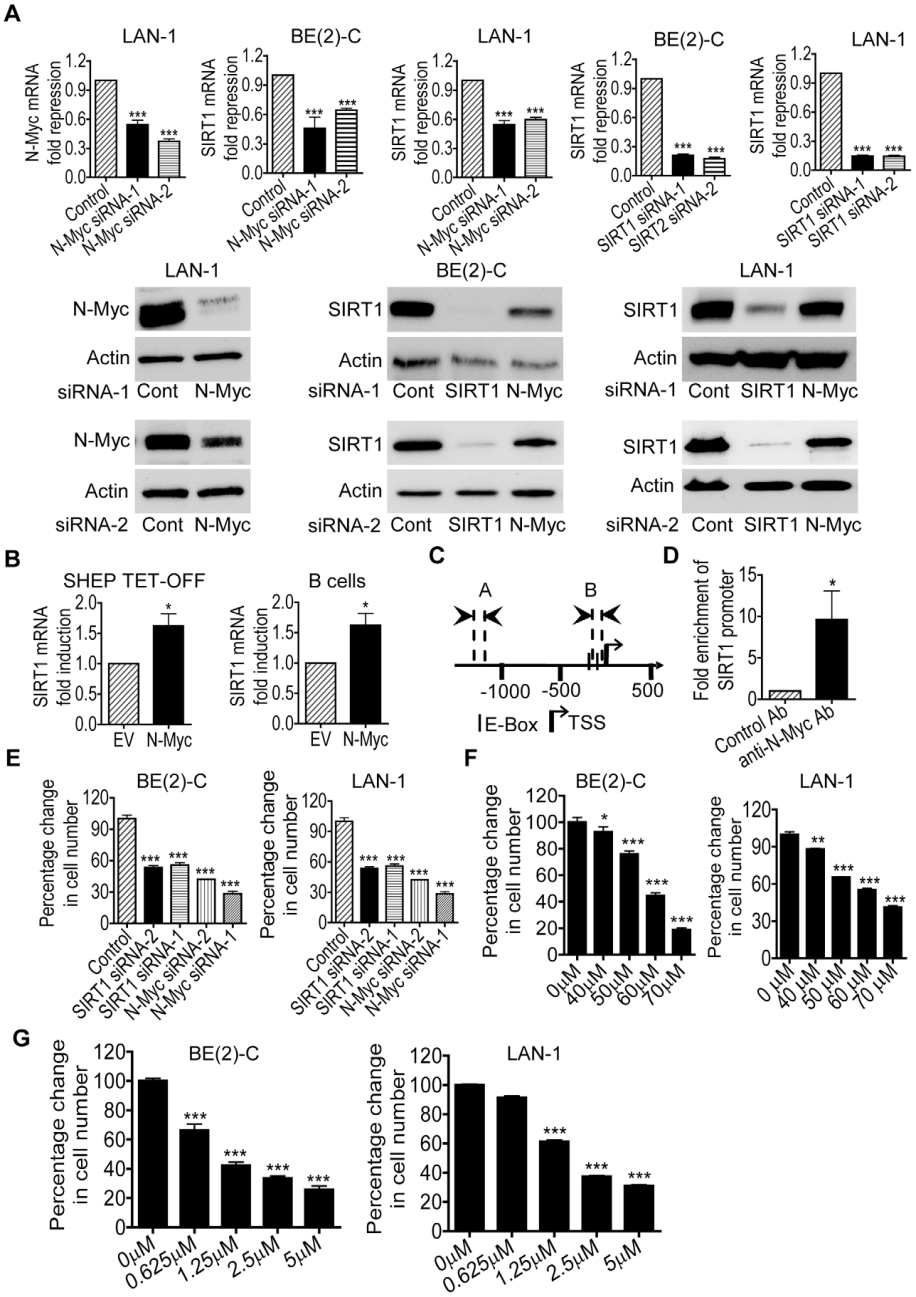
Transcriptional up-regulation of SIRT1 promotes neuroblastoma cell proliferation. (A) BE(2)-C and LAN-1 neuroblastoma cells were transfected with scrambled control (Cont) siRNA, N-Myc siRNA-1, N-Myc siRNA-2, SIRT1 siRNA-1 or SIRT1 siRNA-2 for 48 hours, followed by RNA and protein extraction, real-time RT-PCR and immunoblot analyses of N-Myc and SIRT1 mRNA and protein expression. (B) Tetracycline (TET) was withdrawn from SHEP TET-OFF cell culture medium to induce N-Myc gene expression, and B-cells were purified from normal mouse bone marrow and transfected with a construct over-expressing full-length N-Myc cDNA or empty vector (EV). SIRT1 gene expression in the cells was analysed by real-time RT-PCR. (C) Schematic representation of the human SIRT1 gene promoter. (D) ChIP assay was performed with control or anti-N-Myc antibody (Ab) and primers targeting amplicon B in BE(2)-C cells. Fold enrichment of SIRT1 gene promoter by the antibodies was calculated by dividing the PCR product from antibody-immunoprecipitated samples by the PCR product from input. (E, F, G) BE(2)-C and LAN-1 cells were transfected with scrambled control siRNA, N-Myc siRNA-1, N-Myc siRNA-2, SIRT1 siRNA-1 or SIRT1 siRNA-2 (E), or treated with vehicle control, Cambinol (F) or Tenovin-6 (G). Seventy-two hours later, relative cell numbers were examined by the Alamar blue assay, and expressed as percentage change in cell number. Error bars represented standard error. * indicated *P*<0.05, ** *P*<0.01 and *** *P*<0.001.

By screening human gene promoter regions with GenoMatix software, we found two Myc-responsive element E-boxes −136 bp and −57 bp upstream of the SIRT1 transcription start site. We therefore examined possible modulation of SIRT1 expression by N-Myc. We previously demonstrated that transfection of *MYCN*-amplified BE(2)-C human neuroblastoma cells with N-Myc siRNA No.1 (N-Myc siRNA-1) or No.2 (N-Myc siRNA-2) significantly reduced N-Myc mRNA and protein expression [21]. As shown in Figure 1A, N-Myc siRNA-1 and N-Myc siRNA-2 also significantly reduced N-Myc mRNA and protein expression in *MYCN*-amplified LAN-1 human neuroblastoma cells, and SIRT1 siRNA-1 and SIRT1 siRNA-2 knocked down SIRT1 mRNA and protein expression in both BE(2)-C and LAN-1 cells. Importantly, N-Myc siRNA-1 and N-Myc siRNA-2 significantly reduced SIRT1 mRNA and protein expression in the two neuroblastoma cell lines (Figure 1A). We have previously shown that N-Myc expression was increased by approximately 100% in neuroblastoma SHEP TET-OFF cells, which were stably transfected with a tetracycline withdrawal-inducible N-Myc-expression construct, after tetracycline withdrawal from cell culture medium [22]. As shown in Figure 1B, when N-Myc is over-expressed in SHEP TET-OFF cells after tetracycline withdrawal and in normal mouse bone marrow-derived B cells after transfection with an N-Myc-expression construct (Figure S1), SIRT1 mRNA expression was up-regulated. Chromatin immunoprecipitation (ChIP) assays showed that anti-N-Myc antibody efficiently immunoprecipitated the region of SIRT1 gene core promoter carrying the E-boxes (Figure 1C and 1D). These data suggest that N-Myc up-regulates SIRT1 gene expression by directly binding to the E-Boxes at SIRT1 gene core promoter.

We next examined whether up-regulation of SIRT1 contributed to an N-Myc-induced cancer phenotype. Alamar blue assays revealed that N-Myc siRNA-1, N-Myc siRNA-2, SIRT1 siRNA-1 or SIRT1 siRNA-2 reduced cell numbers by approximately 50% in p53-mutant BE(2)-C and LAN-1 cells in 3 days (Figure 1E). Similarly, repression of SIRT1 with Cambinol, a small molecule SIRT1 inhibitor [23], induced a dose-dependent growth inhibition (Figure 1F). TUNEL assays showed that N-Myc siRNAs, SIRT1 siRNAs and Cambinol did not significantly induce cell death in the p53-mutant neuroblastoma cells (data not shown). Moreover, Alamar blue assays demonstrated that repression of SIRT1 with the small molecule inhibitor Tenovin-6 [24] also induced a dose-dependent growth inhibition (Figure 1G). These data suggest that transcriptional up-regulation of SIRT1 contributes to N-Myc-induced cell proliferation.

### SIRT1 stabilizes N-Myc protein

**Figure 2:**
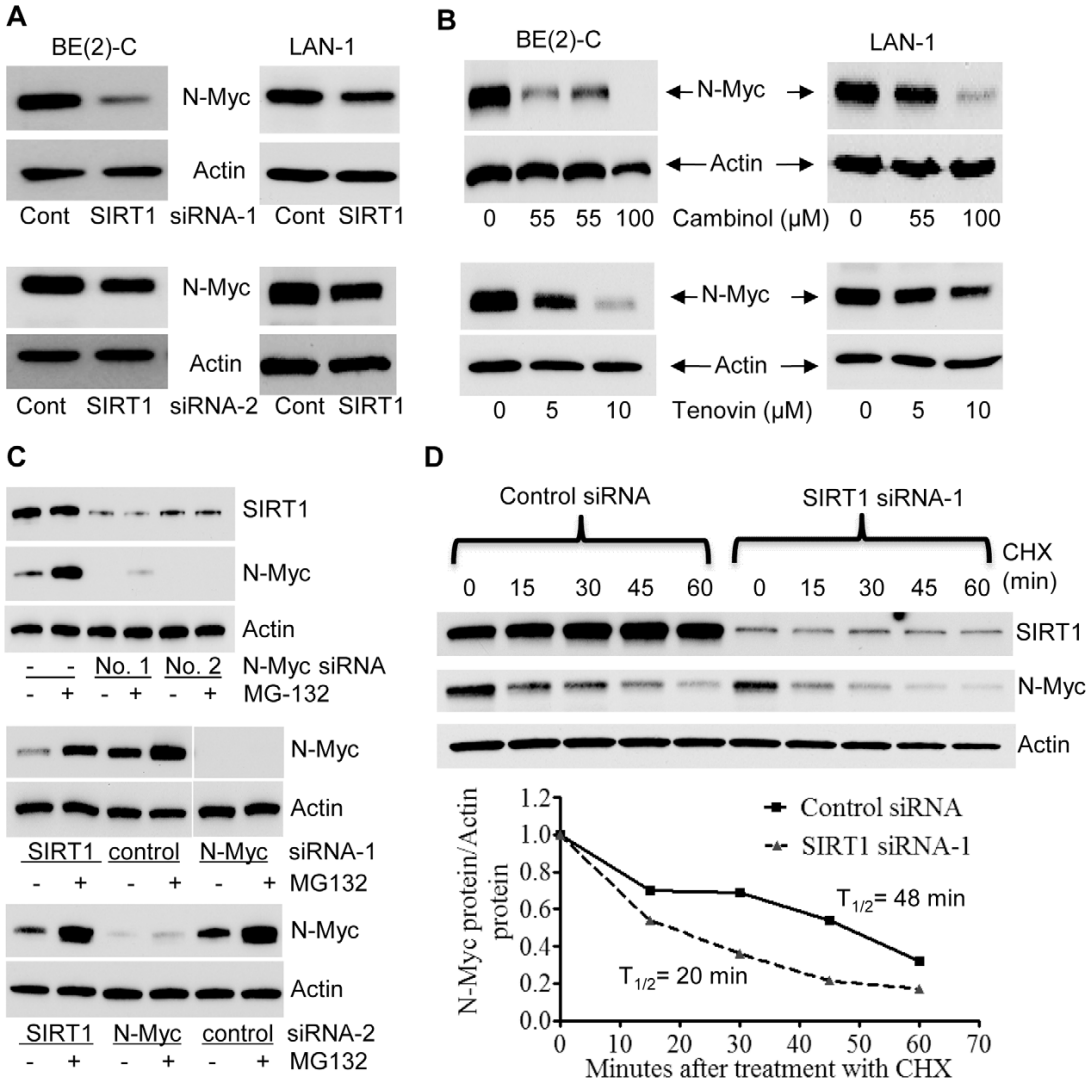
SIRT1 up-regulates N-Myc protein expression by blocking its degradation. (A, B) BE(2)-C and LAN-1 cells were transfected with scrambled control (Cont) siRNA, SIRT1 siRNA-1 or SIRT1 siRNA-2 (A), or treated with the SIRT1 inhibitor Cambinol, Tenovin-6 or vehicle control (B), followed by protein extraction and immunoblot analysis of N-Myc protein. (C) BE(2)-C cells were transfected with scrambled control siRNA, N-Myc siRNA-1, N-Myc siRNA-2, SIRT1 siRNA-1 or SIRT1 siRNA-2 for 48 hours, followed by treatment with the proteasome inhibitor MG-132 (10 µM) for 3 hours. SIRT1 and N-Myc protein expression was analysed by immunoblot. (D) BE(2)-C cells were transfected with scrambled control siRNA or SIRT1 siRNA-1 for 30 hours, and treated with 50 µM cycloheximide (CHX) for the last 0, 15, 30, 45 or 60 minutes. Protein was extracted from the cells and subjected to immunoblot analysis of N-Myc. N-Myc protein level was normalized by actin, the ratio of N-Myc protein and actin protein was artificially set as 1.0 for samples un-treated with CHX, and half life (T_1/2_) of N-Myc protein was obtained from the line chart.

Surprisingly, our immunoblot analyses showed that SIRT1 siRNAs reduced N-Myc protein expression. As shown in Figure 2A, both SIRT1 siRNA-1 and SIRT1 siRNA-2 reduced the N-Myc protein expression level in BE(2)-C and LAN-1 cells. Real-time RT-PCR analysis showed that the SIRT1 siRNAs did not reduce N-Myc mRNA expression (Figure S2A). Moreover, treatment with the SIRT1 inhibitors, Cambinol [23] or Tenovin-6 [24], consistently reduced the expression of N-Myc protein (Figure 2B), but not N-Myc mRNA (Figure S2B).

Because N-Myc protein is degraded through proteasome-mediated proteolysis, we treated BE(2)-C cells with the proteasome inhibitor MG-132 after siRNA transfection. Immunoblot analyses showed that MG-132 dramatically up-regulated the expression of N-Myc protein, but not SIRT1 protein, in cells transfected with scrambled control siRNA (Figure 2C, upper panel). While MG-132 did not increase N-Myc protein expression in cells transfected with N-Myc siRNAs, which ablated N-Myc mRNA, MG-132 significantly up-regulated N-Myc protein expression in cells transfected with SIRT1 siRNA-1 or SIRT1 siRNA-2 for 48 hours (Figure 2C, middle and bottom panels). We next treated BE(2)-C cells with 50 µM cycloheximide (CHX) at different time points after transfection with control siRNA or SIRT1 siRNA-1 for only 30 hours, when the effect of SIRT1 siRNA-1 on N-Myc protein expression was minimal. Immunoblot analysis showed that N-Myc protein half-life was reduced from 48 minutes in cells transfected with control siRNA to 20 minutes in cells transfected with SIRT1 siRNA-1 (Figure 2D). Taken together, these data suggest that SIRT1 reduces proteasome-mediated N-Myc protein degradation and therefore stabilizes N-Myc protein.

### SIRT1 stabilizes N-Myc protein by promoting ERK protein phosphorylation and N-Myc protein phosphorylation at S62

**Figure 3:**
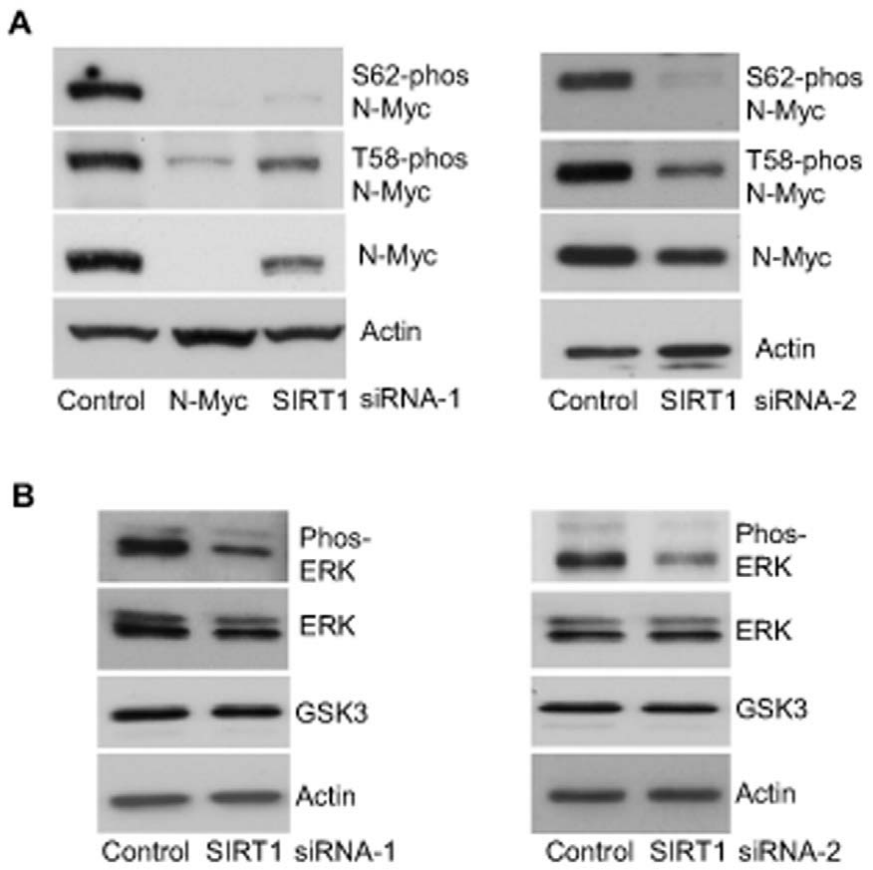
SIRT1 stabilizes N-Myc protein by promoting ERK protein phosphorylation and N-Myc protein phosphorylation at S62. (A, B) BE(2)-C cells were transfected with scrambled control siRNA, SIRT1 siRNA-1 (left panel) or SIRT1 siRNA-2 (right panel), followed by protein extraction. (A) Expression of total N-Myc protein, N-Myc protein phosphorylated at S62 (S62-phos) and N-Myc protein phosphorylated at T58 (T58-phos) was analysed by immunoblot with specific antibodies. (B) Expression of GSK3 protein, total ERK protein and phosphorylated ERK protein (phos-ERK) was analysed by immunoblot with specific antibodies.

When phosphorylated at T58, Myc oncoproteins are degraded through proteasome-mediated proteolysis. By contrast, when phosphorylated at S62, Myc protein degradation is blocked [4], [5]. We therefore examined whether SIRT1 increased N-Myc protein stability by modulating N-Myc protein phosphorylation. As shown in Figure 3A, transfection of BE(2)-C cells with SIRT1 siRNA-1 or SIRT1 siRNA-2 reduced T58-phosphorylated N-Myc protein and total N-Myc protein to a similar extent. However, a much more dramatic reduction in S62-phosphorylated N-Myc was observed after SIRT1 knock-down, suggesting that SIRT1 stabilized N-Myc protein by promoting its phosphorylation at S62.

N-Myc protein phosphorylation at S62 is directly enhanced by phosphorylated ERK [4], and indirectly decreased by glycogen synthase kinase 3 (GSK3) which increases N-Myc protein phosphorylation at T58 and consequent proteasome-mediated degradation [25]. We therefore examined whether SIRT1 modulated ERK protein phosphorylation and GSK3 protein expression. As shown in Figure 3B, SIRT1 siRNA-1 and SIRT1 siRNA-2 had no significant effect on GSK3 protein expression, but consistently decreased ERK protein phosphorylation. These data suggest that SIRT1 stabilizes N-Myc protein by up-regulating ERK protein phosphorylation, which in turn phosphorylates N-Myc protein at S62 and blocks its degradation.

Because SIRT1 siRNA-2 and N-Myc siRNA-2 did not show appreciable differences from SIRT1 siRNA-1 and N-Myc siRNA-1 respectively in all of the above-mentioned experiments, we decided to use SIRT1 siRNA-1 and N-Myc siRNA-1 only in all of the following experiments, and referred them as SIRT1 siRNA and N-Myc siRNA respectively.

### Repression of MKP3 gene expression is required for SIRT1-induced N-Myc protein stabilization and for SIRT1-induced cell proliferation

To identify transcriptional target genes responsible for SIRT1-induced ERK protein phosphorylation, we performed differential gene expression studies with Affymetrix Gene Array in BE(2)-C cells 30 hours after transfection with scrambled control or SIRT1 siRNA. As shown in Dataset S1 and Dataset S2, the gene second most significantly reactivated by SIRT1 siRNA was mitogen-activated protein kinase phosphatase 3 (MKP3)/dual specificity phosphatase 6 (DUSP6)/Pyst1, which selectively de-phosphorylates and inactivates ERK [26], [27]. Importantly, MKP3 was also up-regulated by N-Myc siRNA by approximately 3 fold in BE(2)-C cells 30 hours after siRNA transfection in our previous Affymetrix Gene Array data [21].

**Figure 4:**
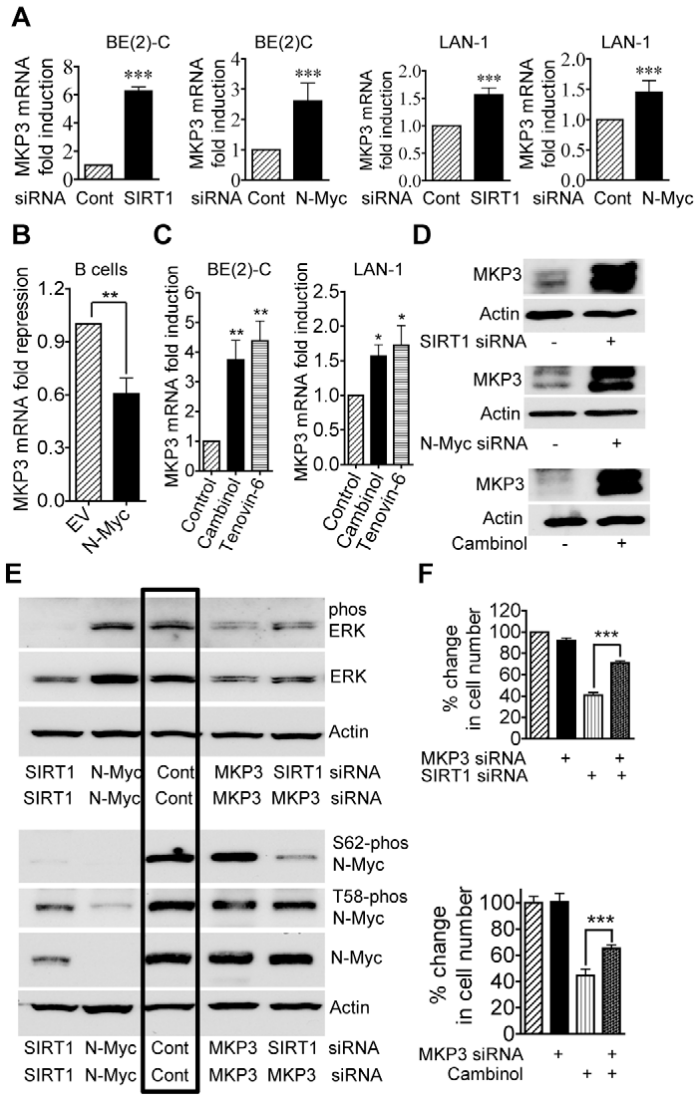
Repression of MKP3 gene expression is required for SIRT1-induced N-Myc protein stabilization and SIRT1-induced cell proliferation. (A, C, D) BE(2)-C and LAN-1 cells were transfected with scrambled control (Cont), SIRT1 siRNA or N-Myc siRNA (A, D), or treated with vehicle control, 55 µM Cambinol or 5 µM Tenovin-6 (C, D). MKP3 mRNA (A, C) and protein (D) expression was analysed by real-time RT-PCR and immunoblot. (B) B cells from normal mouse bone marrow were transfected with a construct over-expressing full-length N-Myc cDNA or empty vector (EV). MKP3 gene expression was analysed by real-time RT-PCR. (E) BE(2)-C cells were transfected with scrambled control siRNA, MKP3 siRNA, SIRT1 siRNA, N-Myc siRNA, or a combination of MKP3 siRNA and SIRT1 siRNA. Phosphorylated ERK, total ERK, S62-phosphorylated (S62-phos) N-Myc, T58-phosphorylated (T58-phos) N-Myc or total N-Myc protein was examined by immunoblot with specific antibodies. (F) BE(2)-C cells were transfected with scrambled control siRNA, MKP3 siRNA, SIRT1 siRNA, or a combination of MKP3 siRNA and SIRT1 siRNA. In separate experiments, BE(2)-C cells were transfected with scrambled control or MKP3 siRNA and treated with vehicle control or 55 µM Cambinol for 72 hours. Relative total numbers of cells were examined by the Alamar blue assay. Error bars represented standard error. *** indicated *P*<0.001.

To validate the gene array data, we performed real-time RT-PCR and immunoblot analyses of MKP3 expression. As shown in Figure 4A and 4D, the expression of MKP3 mRNA and protein was up-regulated by SIRT1 siRNA and N-Myc siRNA in BE(2)-C and LAN-1 cells. Consistently, transfection of primary mouse bone marrow-derived B cells with an N-Myc-expression construct reduced MKP3 expression by approximately 50% (Figure 4B), and repression of SIRT1 with Cambinol or Tenovin-6 reactivated MKP3 expression in both BE(2)-C and LAN-1 cells (Figure 4C and 4D). These data demonstrate that MKP3 is transcriptionally repressed by SIRT1 and N-Myc, and that SIRT1 inhibitors can be applied to reverse the effect. Moreover, RT-PCR analyses demonstrated that both SIRT1 siRNA and the SIRT1 inhibitor Cambinol up-regulated the expression of the other SIRT1 target genes including early growth response 1 (EGR1), Kv channel interacting protein 4 (KCNIP4) and phospholipase C beta 1 (PLCB1), which were randomly selected from the Affymetrix Gene Array data (Dataset S1), in BE(2)-C and LAN-1 cells (Figure S4).

As MKP3 is well-known to selectively de-phosphorylate the ERK protein [26], [27], we examined whether blocking MKP3 gene reactivation could reverse the effects of SIRT1 siRNA on ERK and N-Myc protein de-phosphorylation. As shown in Figure 4E, MKP3 siRNA alone did not have a significant effect on ERK and N-Myc protein phosphorylation, possibly due to a very low basal level of MKP3 expression. While SIRT1 siRNA alone dramatically reduced ERK protein phosphorylation and N-Myc protein phosphorylation at S62, co-transfection with MKP3 siRNA restored phosphorylated ERK, S62-phosphorylated N-Myc and total N-Myc protein levels. These data suggest that SIRT1-modulated transcriptional repression of MKP3 is essential for ERK protein phosphorylation, N-Myc protein phosphorylation at S62 and consequent N-Myc protein stabilization.

We next examined whether transcriptional activation of MKP3 contributed to cell growth inhibition induced by SIRT1 siRNA and the SIRT1 inhibitor Cambinol. While repression of MKP3 gene expression alone did not have an effect on cell proliferation, co-transfection of MKP3 siRNA significantly blocked the growth inhibition caused by SIRT1 siRNA and Cambinol in BE(2)-C (Figure 4F) and LAN-1 (Figure S3) cells. These data indicate that transcriptional repression of MKP3 contributes to SIRT1-induced neuroblastoma cell proliferation.

### SIRT1 and N-Myc repress MKP3 gene transcription by forming a transcriptional repressor complex at Sp1-binding sites of MKP3 gene promoter

**Figure 5:**
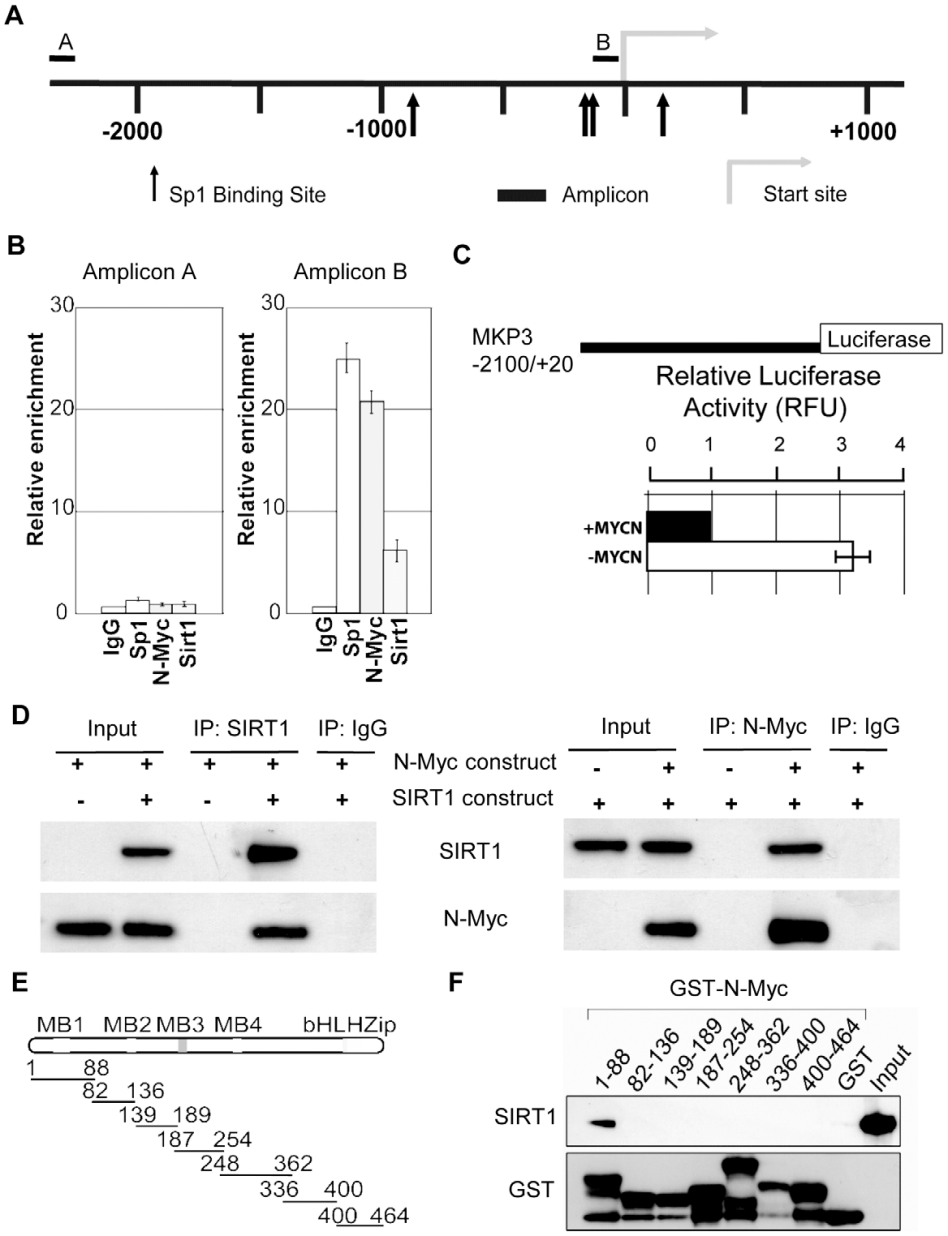
SIRT1 and N-Myc repress MKP3 gene transcription by forming a transcriptional repressor complex at MKP3 gene core promoter. (A) A schematic representation of the MKP3 gene promoter containing the Sp1 binding sites. (B) Dual cross-linking ChIP and quantitative PCR were applied in BE(2)-C cells. Real-time PCR with primers targeting the negative control region (Amplicon A) or the Sp1-binding sites (Amplicon B) were performed. Fold enrichment of MKP3 promoter regions immunoprecipitated by pre-immune serum (IgG), anti-Sp1, anti-N-Myc and anti-SIRT1 antibodies was calculated as the logarithm of the difference between the cycle-threshold obtained with pre-immune serum and the cycle-threshold obtained with the specific antibody. (C) TET-21/N neuroblastoma cells were transfected with a luciferase reporter construct carrying MKP3 gene promoter region. Luciferase activity of the luciferase reporter construct was determined in the presence (- tetracycline) or absence (+ tetracycline) of N-Myc expression, normalized to that of renilla, and expressed as relative fluorescence units (RFU). Error bars represented standard error. *** indicated *P*<0.001. (D) HEK 293 cells were transfected with constructs expressing empty vector, N-Myc and/or SIRT1. Nuclear protein from the cells was immunoprecipitated with an anti-N-Myc, anti-SIRT1 or pre-immune serum (IgG) antibody, and co-immunoprecipitation (IP) products were probed with anti-SIRT1 and anti-N-Myc antibodies by immunoblot. (E) Seven different GST-N-Myc deletion mutant expression constructs were generated. GST-N-Myc proteins carrying different moieties of the full length N-Myc were obtained. MB represents Myc Box, and bHLH-zip the basic helix-loop-helix-zipper region. (F) Immobilized GST-N-Myc proteins were loaded with *in vitro* translated SIRT1 protein. GST-N-Myc complexes were analyzed by immunoblot with an anti-SIRT1 antibody. Amount of loaded GST proteins was also determined by immunoblot.

SIRT1 is known to repress gene transcription by binding to Sp1-binding sites at target gene promoters [28]. We have previously shown that N-Myc represses the transcription of the tissue transglutaminase gene by recruiting HDAC1 protein to tissue transglutaminase gene promoter [22]. As both N-Myc and SIRT1 suppressed MKP3 gene expression, we tested the hypothesis that N-Myc and SIRT1 repressed MKP3 gene transcription by forming a transcriptional repressor complex at Sp1-binding sites of MKP3 gene promoter. Bio-informatics analysis of the MKP3 gene promoter (−2000/+0 from transcription start site) identified one region proximal to the transcription start site enriched for Sp1-binding sites (Figure 5A). Dual cross-linking ChIP assay showed that antibodies against N-Myc, SIRT1 and Sp1 all efficiently immunoprecipitated the region of MKP3 gene promoter carrying Sp1-binding sites (Figure 5B). By contrast, an antibody against Miz1, a protein that is often involved in Myc-driven transcriptional repression [29], immunoprecipitated the gene promoter region of p21 (positive control), but not the gene promoter region of MKP3 (Figure S5A). To confirm that transcriptional suppression of MKP3 was directly mediated by N-Myc, we transfected a Luciferase reporter construct carrying MKP3 gene promoter into TET21/N cells, a human neuroblastoma cell line carrying a *MYCN* transgene under the control of a TET-OFF promoter. Luciferase assays showed that repression of N-Myc expression significantly activated the MKP3 gene promoter (Figure 5C). To demonstrate that N-Myc and SIRT1 form a protein complex, we transfected human embryonic HEK 293 cells with an empty vector, a SIRT1 expressing construct [30] and/or an N-Myc expressing construct, extracted nuclear protein and performed protein co-immunoprecipitation (IP) assays (Figure 5D). Results showed that anti-SIRT1 antibody could efficiently co-immunoprecipitate N-Myc protein, and anti-N-Myc antibody could efficiently co-immunoprecipitate SIRT1 protein. By contrast, anti-SIRT1 antibody did not co-immunoprecipitate Miz1 protein, and anti-Miz1 antibody did not co-immunoprecipitate SIRT1 protein (Figure S5B).

We have previously shown that N-Myc protein binds to the histone deacetylase HDAC1 protein through N-Myc DNA-binding domain [22]. We next sought to determine which domain of N-Myc protein directly interacted with SIRT1. Seven different GST-N-Myc deletion mutant expression constructs were generated (Figure 5E). GST pull-down assay showed that SIRT1 bound only the Myc Box I domain (Figure 5F). Taken together, these findings suggest that SIRT1 forms a transcriptional repressor complex with N-Myc through binding to its Myc Box 1 domain, and that the protein complex represses MKP3 gene transcription by binding to the Sp1-binding sites upstream of MKP3 transcription start site.

### SIRT1 was up-regulated and MKP3 down-regulated in pre-cancerous cells from *TH-MYCN* transgenic mice

**Figure 6:**
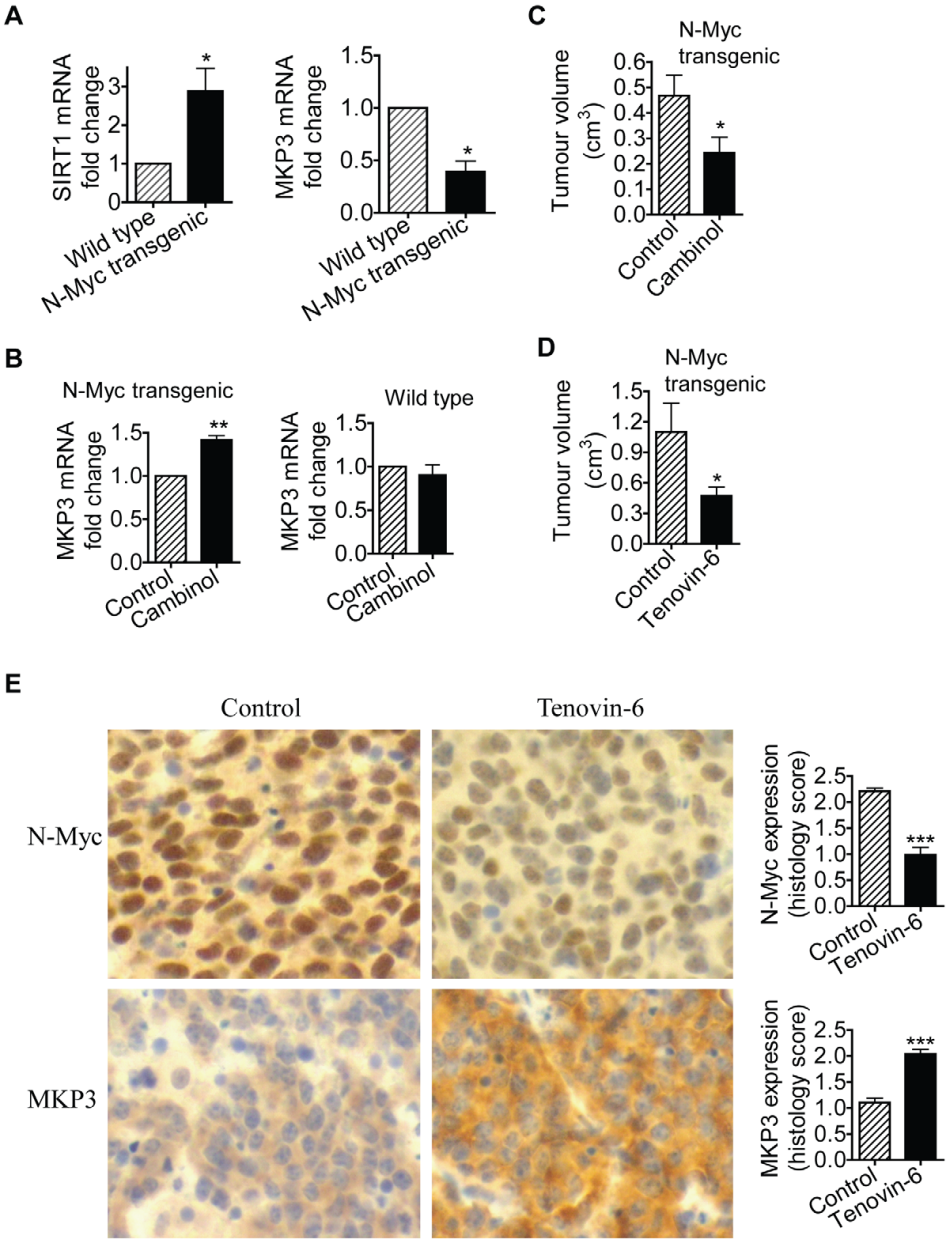
SIRT1 plays an important role in N-Myc–induced neuroblastoma initiation and progression *in vivo*. (A, B) Celiac and superior cervical ganglia were dissected from wild type mice and homozygous *TH-MYCN* transgenic mice at 2 weeks old, and ganglia cells purified. (A) RNA was extracted from the ganglia cells and subjected to real-time RT-PCR analysis of SIRT1 and MKP3 gene expression. SIRT1 and MKP3 expression in ganglia cells from normal mice was artificially set as 1.0. (B) The ganglia cells were treated with vehicle control or 55 µM Cambinol for 24 hours, followed by real-time RT-PCR analysis of MKP3 gene expression. MKP3 gene expression in ganglia cells treated with vehicle control was artificially set as 1.0. (C) Five day old homozygous *TH-MYCN* transgenic mice were injected intra-peritoneally with Cambinol at the dose of 100 mg/kg/day (number  = 8) or vehicle control (number  = 8) for 10 consecutive days. They were then left un-treated for 4 weeks, and sacrificed at the age of 42 days. Tumor volume was measured and analyzed. (D) Forty-eight day old homozygous *TH*-*MYCN* transgenic mice were injected intra-peritoneally with Tenovin-6 at the dose of 50 mg/kg/day (number  = 10) or vehicle control (number  = 10) for 18 consecutive days. The mice were euthanized at the completion of therapy. Tumor volume was measured and analysed. (E) Neuroblastoma tissues from the *TH*-*MYCN* transgenic mice treated with control or Tenovin-6 were examined by immunohistochemistry with anti-N-Myc or MKP3 antibodies and visualized with DAB. The nucleus was counter-stained with haematoxylin. N-Myc and MKP3 protein expression was analysed using the scoring system described in Materials and Methods, and expressed as histology score. Error bars indicated standard error. * indicated *P*<0.05, and ** *P*<0.01.

*TH*-*MYCN* transgenic mice with the *MYCN* oncogene in the germline, driven by the tyrosine hydroxylase (TH) promoter, develop a tumour phenotype which closely resembles human neuroblastoma [31]. We have previously shown that 2-week-old homozygous *TH-MYCN* transgenic mice develop pre-cancerous neuroblast cell hyperplasia in celiac and superior cervical ganglia, which develops into microscopic neuroblastoma in 100% of the mice by 3 weeks of age [32]. In the current investigations, we examined whether N-Myc modulated SIRT1 and MKP3 gene expression in pre-cancerous ganglia cells. As shown in Figure 6A, SIRT1 mRNA expression was increased by 3-fold, and MKP3 gene expression reduced by approximately 60%, in pre-cancerous ganglia cells from 2-week-old *TH-MYCN* transgenic mice, compared with counterpart normal ganglia cells from 2-week-old wild type mice. To test whether SIRT1 modulated MKP3 gene expression in the pre-cancerous cells, we extracted and purified ganglia cells from 2-week-old mice, and treated the cells with vehicle control or Cambinol for 24 hours. As shown in Figure 6B, treatment with Cambinol up-regulated MKP3 gene expression in pre-cancerous ganglia cells from *TH-MYCN* transgenic mice, but not in counterpart normal ganglia cells from wild type mice. These results suggest that N-Myc up-regulates the expression of SIRT1, N-Myc and SIRT1 repress MKP3 gene expression, in pre-cancerous cells during tumor initiation.

### Chemo-prevention with the SIRT1 inhibitor Cambinol suppresses N-Myc–induced neuroblastoma initiation *in vivo*

We then examined whether suppression of SIRT1 activity could partly block tumor initiation *in vivo*. Five day old homozygous *TH-MYCN* transgenic mice were treated with vehicle control or Cambinol daily for 10 consecutive days (before tumor initiation), left un-treated for 4 weeks, and sacrificed at the age of 42 days. As shown in Figure 6C, short-term preventative treatment with Cambinol before tumor initiation significantly reduced tumor volume in *TH-MYCN* transgenic mice four weeks after the discontinuation of Cambinol treatment. The data confirmed the major role of SIRT1 in the initiation of N-Myc-induced neuroblastoma *in vivo*.

### Therapy with the SIRT1 inhibitor Tenovin-6 suppresses N-Myc–induced neuroblastoma progression *in vivo*

Finally, we examined whether suppression of SIRT1 activity impaired the progression of established neuroblastoma *in vivo*. Four-week-old homozygote N-Myc transgenic mice develop palpable neuroblastoma in the abdomen with an incidence of 100% [22]. Cohorts of 20 homozygous N-Myc transgenic mice at the age of 28 days were treated with control or Tenovin-6 daily for 18 days before being euthanized. As shown in Figure 6D, treatment with Tenovin-6 reduced tumor volume by approximately 50% (*P*<0.05) in the N-Myc transgenic mice. Immunohistochemistry analysis showed significantly increased expression of MKP3 protein (*P*<0.001) and decreased expression of N-Myc protein (*P*<0.001) (Figure 6E) in tumour tissues from mice treated with Tenovin-6. These data confirm that SIRT1 plays a major role in the progression of N-Myc-induced neuroblastoma *in vivo*.

## Discussion

SIRT1 gene expression and deacetylase activity are repressed in normal non-malignant cells by tumor suppressors such as p53 [33], hypermethylated in cancer 1 [34] and by the putative tumor suppressor deleted in breast cancer 1 [35], [36]. In this study, we have shown that N-Myc oncoprotein up-regulates SIRT1 gene transcription by directly binding to its gene promoter in neuroblastoma cells, that forced over-expression of N-Myc in normal cells induces SIRT1 gene expression, and that SIRT1 induces neuroblastoma cell proliferation. Moreover, SIRT1 gene expression is up-regulated in pre-cancerous cells from *TH-MYCN* transgenic mice, compared with counterpart normal cells from wild type mice. Taken together, these data suggest that N-Myc oncoprotein is capable of up-regulating SIRT1 gene expression in normal, pre-cancerous and cancer cells, that up-regulation of SIRT1 promotes cell proliferation, and that N-Myc up-regulates SIRT1 gene expression during malignant transformation in pre-cancerous cells. It is worth noting that repression of SIRT1 does not lead to cell death in the neuroblastoma cell lines tested. As the most common mechanism through which SIRT1 blocks cell death is deacetylation of p53 protein, we hypothesize that repression of SIRT1 does not induce significant cell death in BE(2)-C and LAN-1 cells, because p53 is mutated and N-Myc does not modulate cell survival/death in the neuroblastoma cells [21], [22].

The present study has shown that repression of SIRT1 does not affect N-Myc mRNA expression, but reduces ERK protein phosphorylation, N-Myc protein phosphorylation at S62, and consequently enhances proteasome-mediated N-Myc protein degradation. Importantly, we have identified MKP3 as one of the genes most robustly induced by SIRT1 siRNA. We have also shown that repression of MKP3 gene expression blocks the effects of SIRT1 siRNA on ERK protein de-phosphorylation, N-Myc protein de-phosphorylation at S62 and N-Myc protein degradation. Since phosphorylated ERK stabilizes Myc proteins through phosphorylating Myc at S62 [4]–[6] and MKP3 specifically de-phosphorylates and inactivates ERK protein [26], [27], our data suggests that SIRT1 stabilizes N-Myc protein by repressing the expression of MKP3, leading to ERK protein phosphorylation and N-Myc protein phosphorylation at S62. Moreover, our data showing lower expression of MKP3 in pre-cancerous ganglia cells from N-Myc transgenic mice further support this notion. Previously, Otto *et al* have shown that Aurora A stabilizes N-Myc protein by interacting with N-Myc and SCF^Fbxw7^ ubiquitin ligase, and therefore counteracting N-Myc protein ubiquitination and degradation [5]. Our findings reveal a novel pathway through which N-Myc and SIRT1 form a positive feedback loop which represses MKP3 gene expression, leading to ERK protein phosphorylation and consequently N-Myc protein phosphoryaltion at S62 and N-Myc protein stabilization.

SIRT1 is known to repress gene transcription by binding to Sp1-binding sites at target gene promoters [28]. We have previously shown that N-Myc repress the transcription of the tissue transglutaminase gene by recruiting HDAC1 protein to N-Myc DNA-binding domain at gene promoter of tissue transglutaminase [22]. Our present study shows that N-Myc and SIRT1 bind to MKP3 gene core promoter at Sp1-binding sites, repress MKP3 promoter activity and reduce MKP3 gene expression. Our protein co-immunoprecipitation assay reveals that N-Myc and SIRT1 form a protein complex, and GST pull-down assay demonstrates that SIRT1 directly binds to Myc Box 1 domain of N-Myc protein. These data suggest that N-Myc and SIRT1 are contemporaneously bound to form a transcriptional repressor complex at the Sp1-binding sites of MKP3 gene promoter, and consequently repress MKP3 gene transcription. Our data provide the first evidence that a Myc oncoprotein can bind to SIRT1 protein through Myc Box 1 domain, that Myc oncoproteins may possess a more widespread capacity for transcriptional repression by recruiting SIRT1 protein to target gene promoters, and that Myc-mediated transcriptional repression could be reversed by SIRT1 inhibitors.

A number of small molecule inhibitors of class I and II HDACs are currently in clinical trials for the treatment of malignancies of various organ origins [37]. The SIRT1 inhibitor Cambinol and Tenovin-6 have shown promising anti-cancer effects in a range of cancer cell lines and in animal models of Burkitt's lymphoma and skin cancer [23], [24]. In this study, we have found that suppression of SIRT1 with Cambinol or Tenovin-6 re-activates MKP3 gene expression, reduces N-Myc protein level and induces neuroblastoma cell growth arrest. Moreover, Cambinol up-regulates MKP3 gene expression in both neuroblastoma and pre-cancerous cells, but not in counterpart normal cells, preventative therapy with Cambinol reduces tumorigenesis in N-Myc transgenic mice, and therapy with Tenovin-6 reduced tumour progression in neuroblastoma-bearing N-Myc transgenic mice in association with reduced N-Myc protein expression and increased MKP3 protein expression in tumor tissues. Our data suggest that repression of SIRT1 with specific inhibitors, such as Cambinol and Tenovin-6, could be an effective strategy for the prevention and therapy of N-Myc-induced neuroblastoma, and possibly other Myc-induced cancers.

There are currently controversies and debates regarding the role of SIRT1 in cancer. Wang RH *et al* showed that ectopic expression of SIRT1 in BRCA1 mutant breast cancer cells inhibits tumour formation by deacetylating survivin protein [20]. However, in the same study, ectopic expression of SIRT1 in BRCA1 wild type breast cancer cells did not inhibit tumour formation. While SIRT1 was reported to suppress intestinal tumorigenesis in the APC^min/+^ mouse model by deacetylating and inactivating β-catenin [19], a recent study revealed that there was no difference in tumor development when APC*^+/min^* mice crossed with SIRT1-null mice, and that average polyp size was slightly smaller in SIRT1-null APC*^+/min^* mice [38]. Moreover, repression of SIRT1 in APC wild type colon cancer cells induced massive apoptosis in a FOXO4-dependent manner [39]. In the case of prostate cancer, SIRT1 could promote prostatic intraepithelial neoplasia lesion formation through repressing androgen responsive gene expression and consequently inducing autophagy [40]. However, SIRT1 expression is increased in human prostate cancer tissues, compared with adjacent normal prostate tissues [9], [41], and SIRT1 promotes prostate cancer by deacetylating and inactivating FOXO1 protein [41] and by protecting cells against oxidative stress [42]. In addition, SIRT1 is well-known to protect cancer cells against apoptosis by deacetylating p53 [35], [36], Bcl6 [23], FOXO3a [43] and Ku70 [44] in cancer of various organ origins. It is therefore likely that SIRT1 can function either as an oncogene or tumour suppressor, depending on SIRT1 targets in the cellular context, with the dominant target determining the outcome.

Despite the discrepancies with regard to the functional role of SIRT1 in cancer, SIRT1 inhibitors have unanimously shown anti-cancer effects. For example, the SIRT1 inhibitor Melatonin inhibits prostate cancer progression in transgenic adenocarcinoma of the mouse prostate (TRAMP) mice [45], Cambinol partly blocks lymphoma development in nude mice [23], Tenovin-6 suppresses breast cancer and melanoma cell proliferation *in vitro* and blocks melanoma progression in nude mice [24], and Salermide induces dramatic apoptosis in human colon and breast cancer cells [46].

The current study demonstrates that SIRT1 functions as an oncoprotein in N-Myc oncogenesis through forming a transcriptional repressor complex with N-Myc, repressing MKP3 gene transcription and consequently stabilizing N-Myc oncoprotein, and that SIRT1 inhibitors exert anticancer effects against N-Myc-induced neuroblastoma *in vitro* and *in vivo*. It is unlikely that p53 plays a central role in the effects of SIRT1 in neuroblastoma since the BE(2)-C and LAN-1 cells used in our study do not express functional p53 protein due to p53 gene mutation.

In summary, this study demonstrates that a novel pathway, involving transcriptional up-regulation of SIRT1, repression of MKP3 and consequent ERK protein phosphorylation, contributes to N-Myc oncoprotein stability, neuroblastoma cell proliferation and in vivo tumorigenesis. Moreover, the SIRT1 inhibitors reactivate MKP3 gene expression in tumor and pre-cancerous cells, reduce N-Myc protein expression, inhibit N-Myc-induced tumor initiation and progression in vivo. These findings therefore identify SIRT1 as an important co-factor for N-Myc oncogenesis, and provide important evidence for the potential application of SIRT1 inhibitors in the prevention and therapy of N-Myc-induced neuroblastoma.

## Materials and Methods

### Cell culture

Neuroblastoma BE(2)-C, LAN-1, TET-21/N and SHEP TET-OFF cells were cultured in Dulbecco's modified Eagle's medium supplemented with 5% fetal calf serum. Mouse bone marrow-derived B-cells were extracted from mouse bone marrow as described previously [21], and cultured in RPMI 1640 medium supplemented with 10% heat-inactivated fetal calf serum, 50 µM 2-mercaptoethanol and 10 ng/ml recombinant mouse interleukin-7. The animal work was approved by the Animal Care and Ethics Committee of the University of New South Wales, Sydney, Australia.

### siRNA and plasmid transfection

Cells were transfected with plasmid or siRNA (from Qiagen or Ambion) using Lipofectamine 2000 reagent [21].

### RT-PCR and immunoblot analyses

Gene expression in tumor cells was examined by quantitative real-time RT-PCR as described previously [22], [47]. For the analysis of protein expression by immunoblot, cells were lysed, protein extracted and separated by gel electrophoresis. After western transfer, membranes were probed with mouse anti-N-Myc antibody (1∶1000), rabbit anti-SIRT1 antibody (1∶1000), mouse anti-MKP3 antibody (1∶200) (all from Santa Cruz Biotech, CA), mouse anti-phosphorylated ERK (1∶1000), rabbit anti-total ERK (1∶1000) (both from Millipore), mouse anti-total GSK3, rabbit anti-S62 phosphorylated c-Myc (N-Myc) antibody (Abcam, Cambridge, MA) (1∶1000) or rabbit anti-T58 phosphorylated c-Myc (N-Myc) antibody (Abcam) [48], followed by horseradish peroxidase-conjugated anti-mouse (1∶10000) or anti-rabbit (1∶20000) antiserum (Santa Cruz Biotech). Protein bands were visualized with SuperSignal (Pierce, Rockford, IL). The membranes were lastly re-probed with an anti-actin antibody (Sigma) as loading controls.

### Affymetrix gene array study

Neuroblastoma BE(2)-C cells were transfected with scrambled control siRNA, N-Myc siRNA or SIRT1 siRNA. Thirty hours after transfection, RNA was extracted from the cells with RNeasy mini kit. Differential gene expression was examined with Affymetrix GeneChip Gene 1.0 ST Arrays (Affymetrix), according to the manufacturer's instruction. Results from the microarray hybridization were analysed with GeneSpring software (GeneSpring).

### Cell proliferation assay

Cell proliferation was examined with Alamar blue assays [49]. Briefly, cells were plated into 96 well plates, transfected with various siRNAs or treated with different dosages of Cambinol. Seventy-two hours later, cells were incubated with Alamar blue (Invitrogen) for 5 hours, and plates were then read on a micro-plate reader at 570/595 nm. Results were calculated according to the optical density absorbance units and expressed as percentage change in cell number.

### Dual cross-linking ChIP assay

Dual cross-linking ChIP was performed as we previously described [22], with 5 µg control IgG, anti-Sp1 and anti-SIRT1 antibodies. MKP3 promoter region was detected with quantitative PCR with specific primers.

### ChIP assay

ChIP assays were performed with an anti-Miz1 antibody or pre-immune serum (IgG) with samples from BE Miz1-i cells, which were derived from neuroblastoma BE(2)-C cells after stable transfection with a ponasterone-inducible Miz1 expression construct [29]. Binding of Miz1 to MKP3 and p21 (positive control) promoter regions was analysed by quantitative PCR with specific primers.

### Luciferase assay

Modulation of MKP3 gene promoter activity by N-Myc was analysed by luciferase assays. The MKP3 gene promoter construct has been described previously [a kind gift from Dr. J. Licht [50]]. TET-21/N neuroblastoma cells were transiently transfected with the MKP3 gene promoter construct using Lipofectamine 2000 (Invitrogen). Six hours after transfection, medium was replaced and cells were treated with 1 µg/ml tetracycline for 48 hours before Luciferase Assay. Firefly and Renilla activity was measured with a Dual Luciferase Assay kit (Promega, Madison, WI).

### Co-immunoprecipitation assay

Human embryonic HEK 293 cells were transiently transfected with 12 µg of pCMV14-N-Myc, pCDNA3.1-SIRT1 or both with Lipofectamine2000 (Invitrogen) for 36 hours. 0.5 mg of nuclear protein was then incubated overnight with 2 µg of anti-N-Myc, anti-SIRT1 or control IgG antibody. Eluted proteins were immunoblotted with anti-N-Myc or anti-SIRT1 antibody. In separate experiments, HEK293 cells were transiently transfected with 12 µg of pCDNA3.1-SIRT1, pCDNA3.1-Miz1 [29] or both with Lipofectamine2000 (Invitrogen) for 36 hours. 0.5 mg of nuclear protein was then incubated overnight with 2 µg of anti-SIRT1, anti-Miz1 or control IgG antibody. Eluted proteins were immunoblotted with an anti-SIRT1 or anti-Miz1 antibody (Santa Cruz Biotech).

### GST pull-down assay

Seven different GST-N-Myc deletion mutant expression constructs were generated as we described previously [22]. GST-N-Myc proteins were expressed in E.coli, purified and immobilized onto glutathione agarose beads (Sigma). The derived beads were incubated with *in vitro*-translated SIRT1 protein (TNT Quick Coupled Transcription/Translation System, Promega) pre-treated with DNase (GE Healthcare). Purified complexes were analyzed by immunoblot, using an anti-SIRT1 antibody (Sigma).

### Gene expression studies in N-Myc transgenic mice

We have acquired *TH*-*MYCN* transgenic mice from Dr William Weiss [31], and established a stable colony of the mice [21], [22], [32]. Two week old homozygous *MYCN* transgenic mice and matched 2 week old wild type mice from the same hemizygous *MYCN* transgenic mothers were sacrificed. After superior cervical and celiac ganglia were dissected, ganglia cells were purified and cultured as we have described previously [32]. Briefly, celiac and superior cervical ganglia were dissected from mice and placed in Hanks' balanced salt solution (Invitrogen) containing 1 mg/ml collagenase (Sigma) at 4°C for 30 minutes and then dissociated by adding 0.05% trypsin at 37°C for 5 minutes. After washed twice, the samples were re-suspended and triturated in Neurobasal-A media (Invitrogen) supplemented with 0.5 mM L-glutamine, 25 µM glutamic acid and B27 (Invitrogen; 2% vol/vol). Ganglia cells were then cultured in complete Neurobasal-A media on poly-D-lysine and laminin-coated coverslips in 24-well plates and treated with vehicle control or 55 µM Cambinol for 24 hours, followed by RNA extraction and RT-PCR analysis of gene expression. All animal work was approved by the Animal Care and Ethics Committee of the University of New South Wales.

### Chemoprevention of neuroblastoma in N-Myc transgenic mice

Five days old *MYCN* transgenic mice were randomised into two groups, and injected intraperitoneally with Cambinol at the dosage of 100 mg/kg/day or vehicle control once a day for 10 consecutive days. The treatment was then dis-continued for 4 weeks, mice sacrificed at the age of 42 days, and tumor volume measured with a caliph as we described previously [22].

### Experimental therapy of neuroblastoma in *TH-MYCN* transgenic mice

Four-week-old homozygous *TH-MYCN* transgenic mice develop spontaneous abdominal neuroblastoma with an incidence of 100%. Twenty-eight day old *TH-MYCN* transgenic mice were randomised into two groups, and injected intra-peritoneally with Tenovin-6 at the dosage of 50 mg/kg/day or vehicle control [24] once a day for 18 consecutive days. At the completion of the therapy, the mice were euthanized, tumors collected, tumor volume measured with a caliph as we described previously [22], and tumor tissues paraffin-embedded.

### Immunohistochemistry studies

Mouse tissue sections were de-paraffinised, rehydrated, blocked with 3% hydrogen peroxide and serum. Mouse anti-N-Myc antibody (1∶200) and mouse anti-MKP3 antibody (1∶100) were biotinylated with an Animal Research Kit (DakoCytomation, Glostrup, Denmark), according to the manufacturer's instructions. Tumour sections were incubated with the biotinylated mouse anti-N-Myc antibody or mouse anti-MKP3 antibody and then streptavidin-horseradish peroxidase, and visualized with diaminobenzidine (DAB) solution (DakoCytomation). The cell nucleus was counterstained with haematoxylin. Analyses of the immunohistochemistry staining were performed using our previously established scoring system [51]. Briefly, high level of expression of N-Myc and MKP3 was defined as positive staining with intensity 3+ in >33% of cells; moderate-high staining was defined as intensity 2+ in >33% of positive staining, up to intensity 3+ in 33% of cells; and low expression was defined as any staining with 1+ intensity, up to intensity 2+ in 33% of cells.

### Statistical analysis

All experiments were repeated for at least 3 times in duplicates. All data for statistical analysis were calculated as mean ± standard error. Differences were analyzed for significance using ANOVA among groups or unpaired t-test for two groups. A probability value of 0.05 or less was considered significant.

## Supporting Information

Figure S1 N-Myc is over-expressed in normal mouse bone marrow-derived B cells after transfection with a construct encoding full-length N-Myc cDNA. Mouse bone marrow-derived B cells were purified from normal mouse bone marrow and transfected with a construct encoding full-length N-Myc cDNA or empty vector (EV) for 48 hours. N-Myc expression in the cells was analysed by real-time RT-PCR. N-Myc expression in cells transfected with EV was artificially set as 1.0.
(PDF)

Figure S2 Repression of SIRT1 does not consistently decrease N-Myc gene expression. (A) Neuroblastoma BE(2)-C and LAN-1 cells were transfected with scrambled control siRNA, SIRT1 siRNA-1 or SIRT1 siRNA-2 for 48 hours. (B) BE(2)-C and LAN-1 cells were treated with vehicle control, 55 µM Cambinol or 5 µM Tenovin-6 for 48 hours. Gene expression of N-Myc was analysed by real-time RT-PCR. N-Myc gene expression in control siRNA-transfected samples (A) or vehicle control-treated samples (B) was artificially set as 1.0.
(PDF)

Figure S3 Repression of MKP3 contributes to SIRT1-induced cell proliferation. (A) LAN-1 cells were transfected with scrambled control siRNA, MKP3 siRNA, SIRT1 siRNA, or combination of MKP3 siRNA and SIRT1 siRNA. (B) LAN-1 cells were transfected with scrambled control or MKP3 siRNA and treated with vehicle control or Cambinol for 72 hours. (A, B) Relative total numbers of cells were examined by the Alamar blue assay, measured as optical density (OD) units of absorbance, and expressed as a percentage change of absorbance for the experimental samples, over that for control samples transfected with scrambled control siRNA (A) or treated with vehicle (B). Error bars represented standard error. *** indicated *P*<0.001.
(PDF)

Figure S4 SIRT1 siRNA and the SIRT1 inhibitor Cambinol reactivate the expression of the same set of genes. Neuroblastoma BE(2)-C and LAN-1 cells were transfected with scrambled control siRNA or SIRT1 siRNA-1, or treated with Cambinol at the concentration of 100 µM, 55 µM (IC_50_ for inhibiting SIRT1) or 0 µM (vehicle control) for 48 hours. Gene expression of EGR1 (A), KCNIP4 (B) and PLCB1 (C) was analysed by real-time RT-PCR. Gene expression in control siRNA-transfected samples or vehicle control-treated samples was artificially set as 1.0. Error bars represented standard error. * indicated *P*<0.05, ** *P*<0.01 and *** *P*<0.001.
(PDF)

Figure S5 Miz1 is not required for transcriptional repression of MKP3. (A) BE Miz1-i cells, which were derived from neuroblastoma BE(2)-C cells after stable transfection with a ponasterone-inducible Miz1 expression construct, were used. ChIP assays were performed in BE Miz1-i cells with or without ponasterone treatment to determine the association of Miz1 to MKP3 gene promoter and p21 gene promoter (positive control). Region A represented the promoter region distal from the transcription start site, and region B the promoter region surrounding the transcription start site. Relative enrichment of a given promoter region obtained with a specific antibody was compared with that obtained with pre-immune serum (IgG), which was set to 1 in the graph. Results are the mean ± standard error of 4 independent experiments. (B) HEK 293 cells were transfected with constructs expressing empty vector, SIRT1 and/or Miz1. Nuclear protein from the cells was immunoprecipitated with an anti-SIRT1, anti-Miz1 or pre-immune serum (IgG) antibody, and co-immunoprecipitation (IP) products were probed with anti-SIRT1 and anti-Miz1 antibodies by immunoblot.
(PDF)

Dataset S1 Genes up-regulated by SIRT1 siRNA-1 by more than 2 fold, as identified by Affymetrix gene array analysis, in neuroblastoma BE(2)-C cells 30 hours after transfection with SIRT1 siRNA-1 or control siRNA.
(XLS)

Dataset S2 Genes down-regulated by SIRT1 siRNA-1 by more than 2 fold, as identified by Affymetrix gene array analysis, in neuroblastoma BE(2)-C cells 30 hours after transfection with SIRT1 siRNA-1 or control siRNA.
(XLS)
