# Supplementary figures and images for: SIRT1 Promotes N-Myc Oncogenesis through a Positive Feedback Loop Involving the Effects of MKP3 and ERK on N-Myc Protein Stability
Source: PLoS Genet. 2011 Jun 16;7(6):e1002135. doi: 10.1371/journal.pgen.1002135 (PMC3116909; doi:10.1371/journal.pgen.1002135)

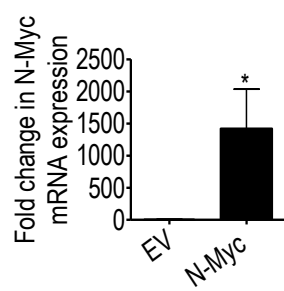

Supplement: Figure S1 — N-Myc is over-expressed in normal mouse bone marrow-derived B cells after transfection with a construct encoding full-length N-Myc cDNA. Mouse bone marrow-derived B cells were purified from normal mouse bone marrow and transfected with a construct encoding full-length N-Myc cDNA or empty vector (EV) for 48 hours. N-Myc expression in the cells was analysed by real-time RT-PCR. N-Myc expression in cells transfected with EV was artificially set as 1.0. (PDF) [file pgen.1002135.s001.pdf]

**A**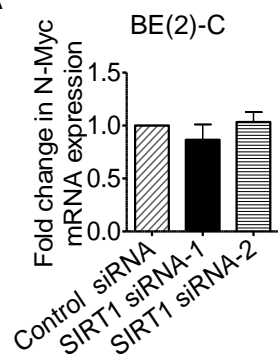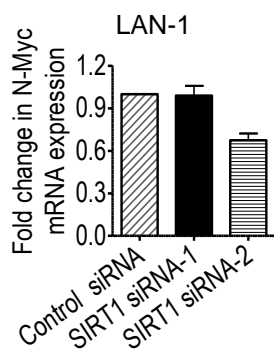**B**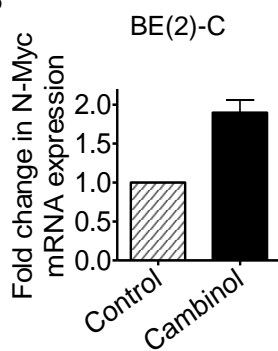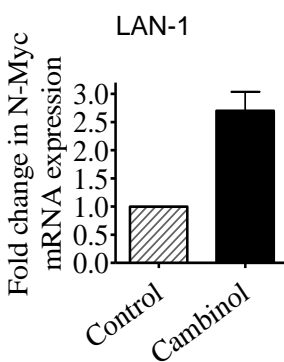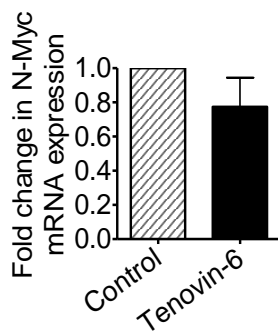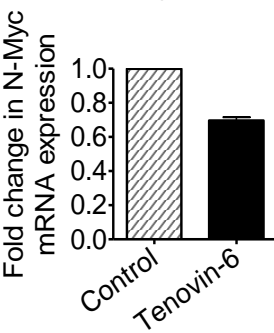

Supplement: Figure S2 — Repression of SIRT1 does not consistently decrease N-Myc gene expression. (A) Neuroblastoma BE(2)-C and LAN-1 cells were transfected with scrambled control siRNA, SIRT1 siRNA-1 or SIRT1 siRNA-2 for 48 hours. (B) BE(2)-C and LAN-1 cells were treated with vehicle control, 55 µM Cambinol or 5 µM Tenovin-6 for 48 hours. Gene expression of N-Myc was analysed by real-time RT-PCR. N-Myc gene expression in control siRNA-transfected samples (A) or vehicle control-treated samples (B) was artificially set as 1.0. (PDF) [file pgen.1002135.s002.pdf]

**A**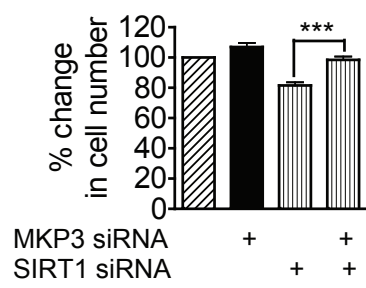**B**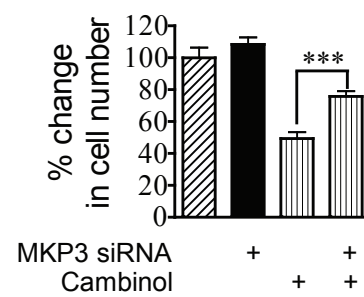

Supplement: Figure S3 — Repression of MKP3 contributes to SIRT1-induced cell proliferation. (A) LAN-1 cells were transfected with scrambled control siRNA, MKP3 siRNA, SIRT1 siRNA, or combination of MKP3 siRNA and SIRT1 siRNA. (B) LAN-1 cells were transfected with scrambled control or MKP3 siRNA and treated with vehicle control or Cambinol for 72 hours. (A, B) Relative total numbers of cells were examined by the Alamar blue assay, measured as optical density (OD) units of absorbance, and expressed as a percentage change of absorbance for the experimental samples, over that for control samples transfected with scrambled control siRNA (A) or treated with vehicle (B). Error bars represented standard error. *** indicated P<0.001. (PDF) [file pgen.1002135.s003.pdf]

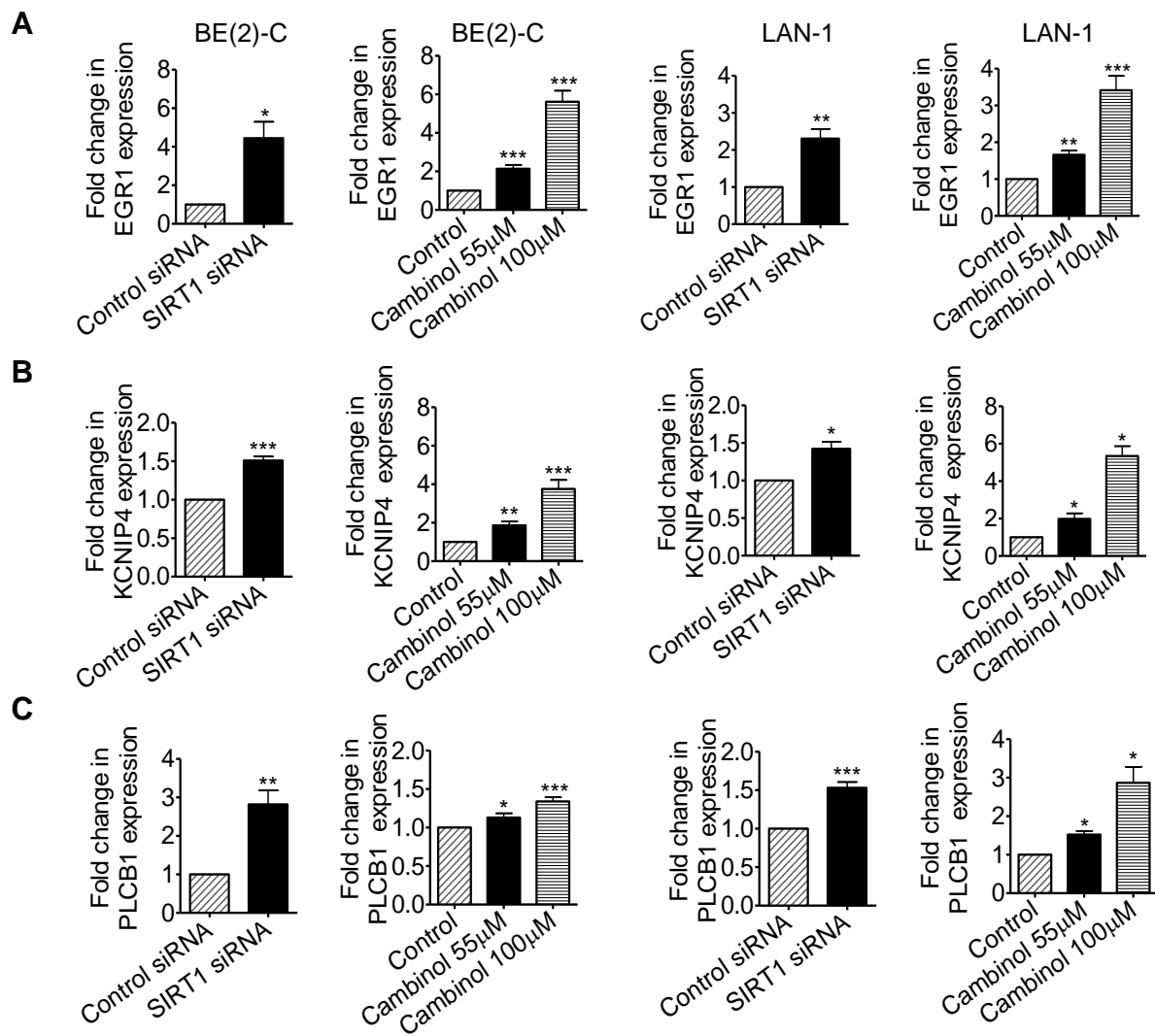

Supplement: Figure S4 — SIRT1 siRNA and the SIRT1 inhibitor Cambinol reactivate the expression of the same set of genes. Neuroblastoma BE(2)-C and LAN-1 cells were transfected with scrambled control siRNA or SIRT1 siRNA-1, or treated with Cambinol at the concentration of 100 µM, 55 µM (IC50 for inhibiting SIRT1) or 0 µM (vehicle control) for 48 hours. Gene expression of EGR1 (A), KCNIP4 (B) and PLCB1 (C) was analysed by real-time RT-PCR. Gene expression in control siRNA-transfected samples or vehicle control-treated samples was artificially set as 1.0. Error bars represented standard error. * indicated P<0.05, ** P<0.01 and *** P<0.001. (PDF) [file pgen.1002135.s004.pdf]

**A**

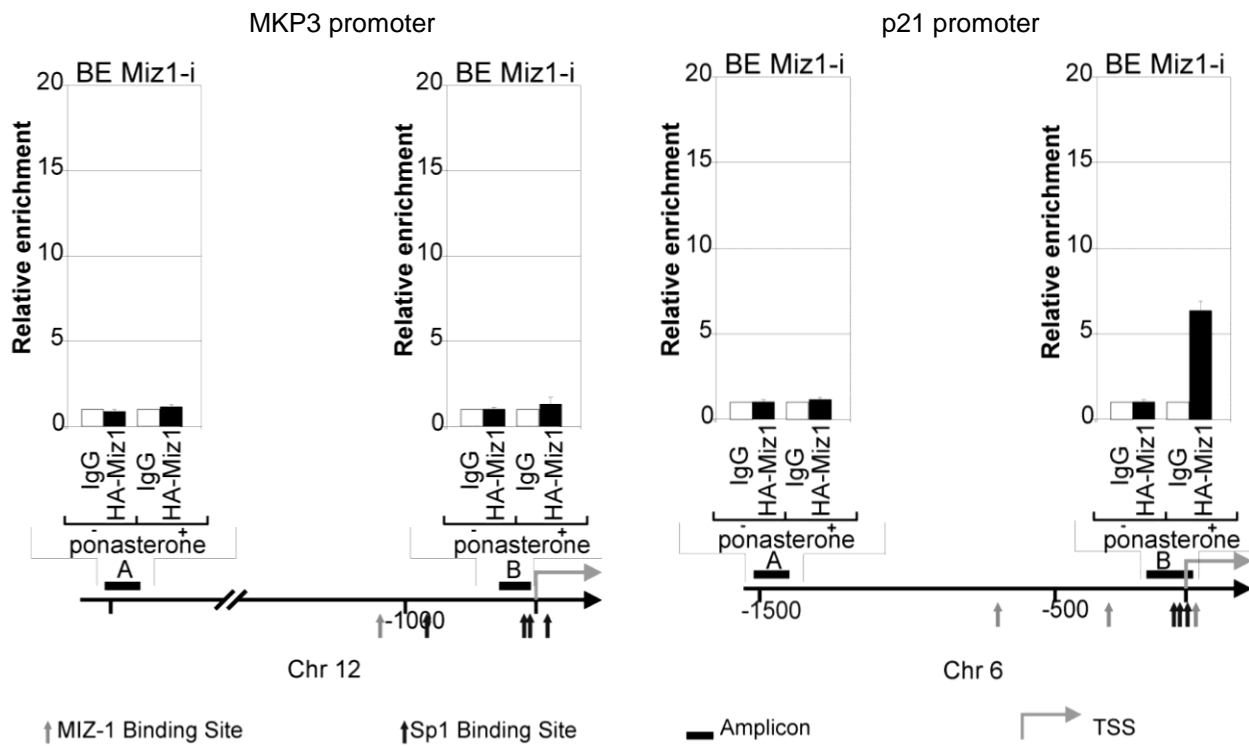

# B

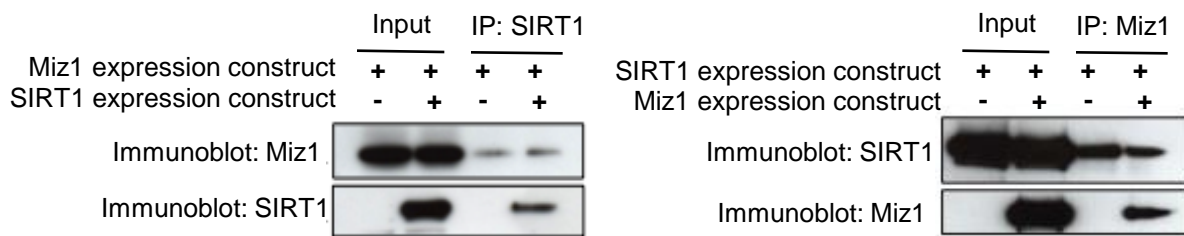

Supplement: Figure S5 — Miz1 is not required for transcriptional repression of MKP3. (A) BE Miz1-i cells, which were derived from neuroblastoma BE(2)-C cells after stable transfection with a ponasterone-inducible Miz1 expression construct, were used. ChIP assays were performed in BE Miz1-i cells with or without ponasterone treatment to determine the association of Miz1 to MKP3 gene promoter and p21 gene promoter (positive control). Region A represented the promoter region distal from the transcription start site, and region B the promoter region surrounding the transcription start site. Relative enrichment of a given promoter region obtained with a specific antibody was compared with that obtained with pre-immune serum (IgG), which was set to 1 in the graph. Results are the mean ± standard error of 4 independent experiments. (B) HEK 293 cells were transfected with constructs expressing empty vector, SIRT1 and/or Miz1. Nuclear protein from the cells was immunoprecipitated with an anti-SIRT1, anti-Miz1 or pre-immune serum (IgG) antibody, and co-immunoprecipitation (IP) products were probed with anti-SIRT1 and anti-Miz1 antibodies by immunoblot. (PDF) [file pgen.1002135.s005.pdf]
